# Use of Adipose-Derived Stem Cells to Support Topical Skin Adhesive for Wound Closure: A Preliminary Report from Animal In Vivo Study

**DOI:** 10.1155/2016/2505601

**Published:** 2016-10-10

**Authors:** Maciej Nowacki, Katarzyna Pietkun, Arkadiusz Jundziłł, Tomasz Kloskowski, Dariusz Grzanka, Joanna Skopinska-Wisniewska, Kinga Scibior, Maciej Gagat, Marta Pokrywczyńska, Alina Grzanka, Wojciech Zegarski, Rafał Czajkowski, Tomasz Drewa, Barbara Zegarska

**Affiliations:** ^1^Chair and Department of Surgical Oncology, Ludwik Rydygier Collegium Medicum in Bydgoszcz, Nicolaus Copernicus University in Torun, Oncology Centre-Prof. Franciszek Łukaszczyk Memorial Hospital in Bydgoszcz, ul. Romanowskiej 2, 85-796 Bydgoszcz, Poland; ^2^Chair of Cosmetology and Aesthetic Dermatology, Ludwik Rydygier Collegium Medicum in Bydgoszcz, Nicolaus Copernicus University in Torun, ul. Jagiellońska 15, 85-067 Bydgoszcz, Poland; ^3^Chair and Department of Rehabilitation, Ludwik Rydygier Collegium Medicum in Bydgoszcz, Nicolaus Copernicus University in Torun, ul. M. Curie Skłodowskiej 9, 85-094 Bydgoszcz, Poland; ^4^Department of Plastic, Reconstructive and Aesthetic Surgery, Ludwik Rydygier Collegium Medicum in Bydgoszcz, Nicolaus Copernicus University in Torun, ul. M. Curie Skłodowskiej 9, 85-094 Bydgoszcz, Poland; ^5^Chair of Urology, Department of Regenerative Medicine, Ludwik Rydygier Collegium Medicum in Bydgoszcz, Nicolaus Copernicus University in Torun, ul. M. Curie Skłodowskiej 9, 85-094 Bydgoszcz, Poland; ^6^Department and Clinic of Dermatology, Sexually Transmitted Diseases and Immunodermatology, Ludwik Rydygier Collegium Medicum in Bydgoszcz, Nicolaus Copernicus University in Torun, ul. M. Curie Skłodowskiej 9, 85-094 Bydgoszcz, Poland; ^7^Department of Chemistry of Biomaterials and Cosmetics, Faculty of Chemistry, Nicolaus Copernicus University in Torun, Gagarina 7, 87-100 Torun, Poland; ^8^Department of Histology and Embryology, Ludwik Rydygier Collegium Medicum in Bydgoszcz, Nicolaus Copernicus University in Torun, ul. Karłowicza 24, 85-092 Bydgoszcz, Poland

## Abstract

The aim of this study was to determine the local and systemic effects of adipose-derived stem cells (ADSCs) as a component of topical skin adhesive in an animal artificial wound closure model. In presented study the cosmetic effects, histological analysis, mechanical properties, and cell migration have been assessed to evaluate the usefulness of ADSCs as supporting factor for octyl blend cyanoacrylate adhesive. The total of 40 rats were used and divided into six groups. In the Study Group, ADSCs were administered by multipoint injection of the six surrounding intrawound areas with additional freely leaving procedure of the cells between the skin flaps just before applying adhesive to close the wound. Five control groups without using ADSCs, utilizing different types of standard wound closure, were created in order to check efficiency of experimental stem cell therapy. In our study, we proved that ADSCs could be used effectively also as a supportive tool in topical skin adhesive for wound closure. However we did not achieve any spectacular differences related to such aspects as better mechanical properties or special biological breakthroughs in wound healing properties. The use of stem cells, especially ADSCs for wound closure can provide an inspiring development in plastic and dermatologic surgery.

## 1. Introduction

Topical skin adhesives, popularly known as tissue or skin glue, are increasingly used as wound closure devices [1–5]. The need for such products is still growing, observable in the systematically expanded use of adhesives for procedures such as port-incision wound closing after laparoscopic surgeries or the growing list of novel uses of topical adhesives such as in the prevention of sternal-wound infection in cardiac surgery [6]. Until now, several in vitro and in vivo studies have been performed to develop the most effective type of adhesive or to establish the optimal method of their use [7]. Those tests were performed mainly because, for many years, the best-known and most used topical adhesives were based on short-chained and long-chained cyanoacrylates, with the most representative octyl-2-cyanoacrylate adhesive, and were still not ideal [8]. The first cyanoacrylate adhesive for skin closure was developed in 1949 and since this date this type of adhesive is supplied as monomers in a liquid form [9]. But the octyl-2-cyanoacrylate adhesive (OCA), also known as “dermabond,” was firstly approved by FDA scarcely in 1998. This time period of fast 50 years of limited clinical use of skin adhesives was considered mainly with previous problems of other forms of cyanoacrylates than OCA to achieve proper tensile strength [10]. Generally the standard skin adhesives self-polymerize to join the edges of wound and to aid its cicatrisation [11]. The current search for an ideal topical adhesive strives mainly for such features as faster wound or incision healing, absence of inflammatory or foreign-body response, optimal quality of microbial barrier, maximum bursting strength, and, the most important from patients' viewpoint, a generally aesthetic outcome [12].

Among mesenchymal stem cells, in dermatology the most commonly used are the adipose-derived stem cells (ADSCs) [13]. Fat tissue is an excellent source of mesenchymal stem cells; they can be obtained during minimally invasive cosmetic liposuction; procedure is characterized by low rate of donor-site complications [14]. Isolation procedure of ADSCs is very simple, and it is characterized with high efficiency, 100–1000 times greater in cellular yield compared to bone-marrow MSCs [15]. ADSCs are mesodermal origin and have potential to differentiate into many cell types also from ectodermal and endodermal lineage. Regeneration process induced by ADSCs could be also induced by its paracrine effect releasing cytokines and growth factors [16]. The positive influence of ADSCs as a supporting tool in procedures in the fields of aesthetic dermatology and plastic surgery has been proven in basic and preclinical studies [17]. Use of autologous transplantation of ADSCs showed promising properties in full-thickness graft survival, scar reduction, and wound healing [18]. Stem cells were used alone or together with scaffold or growth factors. Such therapy resulted in wound healing enhancement, angiogenesis induction, increasing endothelial cells recruitment, and stimulation of collagen neosynthesis which is increasing dermal thickness [19–21]. In some studies, authors suggested that hydrogels composed of sodium alginate, gum Arabic, and calcium ions could be used effectively with ADSCs for wound healing, but such solutions, aside from being nontoxic, are still not fully effective and are problematic to use in any translational projects on a wider scale [22]. For our knowledge this is the first work describing connection of ADSCs and topical skin adhesives for wound healing.

The aim of this study was to determine the local and systemic effects of adipose-derived stem cells as a component of topical skin adhesive in an animal artificial wound closure model. In presented study the cosmetic effects, histological analysis, mechanical properties, and cell migration have been assessed to evaluate the usefulness of ADSCs as supporting factor for octyl blend cyanoacrylate adhesive.

## 2. Materials and Methods

### 2.1. Animals

Forty nude athymic RNU (Crl:NIH-Foxn1^rnu^) rats were used in the study. Thirty-five animals from this group were used directly in in vivo experiments; the remaining five animals were used as donors of ADSCs. All animals were derived from the Charles River strain. All experiments were approved by and conducted in accordance with the local ethical committee (approval number 34/2014). All procedures included assurance of proper breeding conditions and animal welfare and were conducted in accordance with the guidelines of the European Union (Directive 2010/63/EU) and recommendations of the National Research Council. Animals were divided into six groups. The first group, containing 10 animals, was the study group (Study Group); the other five groups contained five animals each and constituted control groups CI, CII, CIII, CIV, and CV. In the Study Group, after a previously performed incision, ADSCs were combined with adhesive application for wound closure. In control group C1, only adhesive was used. In control group C2, adhesive was combined with Nexcare™ Steri-Strip™ closures. In control group C3, we used only Nexcare Steri-Strip closures, and in C4 group, the wounds were closed using only sutures. In group C5, no closing material was applied at all.

### 2.2. ADSC Isolation, Culture, and Preparation

Adipose-derived stem cells were isolated from material obtained from the retroperitoneal space of donor rats. Briefly, adipose tissue (1 g of tissue in 1 mg/mL of enzyme) was digested in collagenase type I solution (Sigma-Aldrich, Germany) for 30 minutes at 37°C with shaking. The reaction was stopped by the addition of Dulbecco's modified Eagle's medium (DMEM/F12; HyClone, USA) supplemented with 10% fetal bovine serum (FBS; PAN-Biotech, Germany) and antibiotics. The cell suspension was next filtered through a 100-*μ*m cell strainer (BD Biosciences, USA) and then centrifuged at 350 ×g for 5 minutes. Obtained cells were seeded on a 25-cm^2^ flask and cultured in DMEM/F12 medium supplemented with 10% FBS, fibroblast growth factor (10 ng/mL; Sigma-Aldrich, Germany), penicillin with streptomycin (100 *μ*g/mL, HyClone, USA), and amphotericin B (5 *μ*g/mL, Corning, USA) at 37°C, 5% CO_2_, and 95% humidity until the third passage. Phenotype and differentiation potential of ADSCs were confirmed earlier [23]. Before implantation, ADSCs were labeled with the fluorochrome PKH26 (Sigma-Aldrich, Germany) to assess migration after cell injection.

### 2.3. Creation and Closing of Artificial Wounds

**Figure 1:**
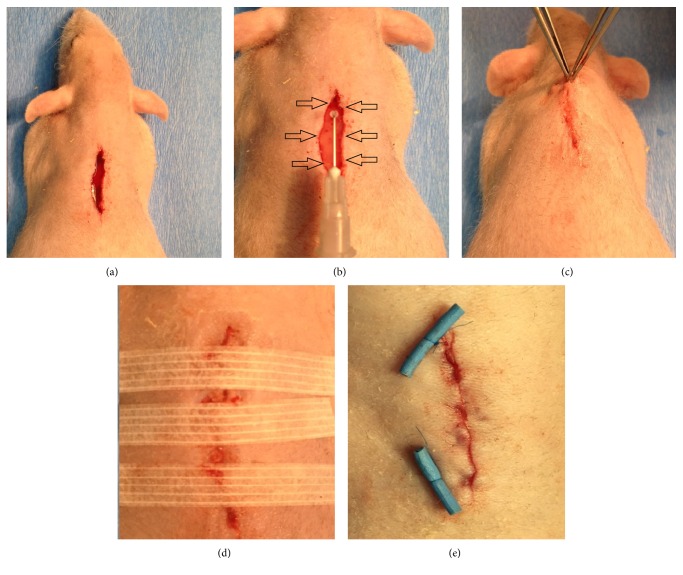
Creation of artificial wounds and application of different wound-closing procedures. (a) The artificial, 1.5-cm, vertical incision using standard surgical scalpel (blade number 24, Swann Morton, UK) made in dorsal region. (b) The ADSC cells administrated in multipoint injection of six surrounding intrawound areas, with additional freely leaving procedure of the cells between skin flaps just before applying adhesive (Indermil flexifuze, Connexicon Medical Ltd., Ireland) to close the wound. (c) Rat from C1 group; only adhesive and delicate approximation of skin surfaces using anatomical forceps was employed. (d) Presentation of animal from the C3 group, in which only Steri-Strips were used. (e) C4 group: wounds were closed with subcuticular running sutures with 7-0 polypropylene sutures (Prolene Ethicon, Johnson & Johnson, USA) and secured on the ends with mild rubber separators.

After anesthesia, a large, 1.5-cm vertical incision was made using a standard surgical scalpel (blade number 24, Swann Morton, UK) in the dorsal region (Figure 1(a)). This location was selected to prevent self-wound irritation; the skin was previously completely disinfected using octenisept® (Schülke & Mayr GmbH., Germany). Immediately after performing the artificial wounds, animals from all groups underwent wound closing, depending on the chosen method. In Study Group, ADSCs were used together with adhesive to close the wound. According to current knowledge, it is not possible to suspend the previously cultivated and prepared stem cells safely in adhesive. Cells suspended in a very small amount of medium cause a very fast bonding process and very rapid crystallization of the entire volume of octyl-2-cyanoacrylate adhesive. That is why, in this study, the ADSCs were administered by multipoint injection of the six surrounding intrawound areas and freely leaving the cells between the skin flaps just before the adhesive (Indermil® flexifuze™, Connexicon Medical Ltd., Ireland) application (Figure 1(b)). In control group C1, only adhesive and delicate approximation of skin surfaces with anatomical forceps were used (Figure 1(c)). In control group C2, adhesive was used with 3 × 75 mm Steri-Strip closures (3M Nexcare™ Steri-Strips™; USA) by a transversal setting of three strips according to the standard guidelines and recommendations used in plastic surgery and dermatosurgery. In control group C3, only Steri-Strips were used (Figure 1(d)); in control group C4, the wounds were closed with subcuticular running suturing with 7-0 polypropylene sutures (Prolene™ Ethicon, Johnson & Johnson, USA) and secured on the ends with mild rubber separators (Figure 1(e)). The sutures were removed in this group always one week after experimental procedure. In group C5, no closing material was applied, and the bleeding open wound was left open.

### 2.4. Assessment of Cosmetic Effects

Cosmetic effect assessment was performed twice. First, after 1.5 months, the wound healing process in all animals was assessed macroscopically after sedation. The second assessment was performed at 3 months, the end of the study, just after all animals were sacrificed by anesthesia overdose with ketamine and xylazine (Sedazin and Bioketan, Biowet, Puławy, Poland). The monitoring period was selected to maximize detection of PKH26 fluorescence, a marker used in cell migration assay, which has a half-life in vivo of approximately 100 days. This period is sufficient for in vivo studies in the field of experimental aesthetic dermatology and plastic surgery [17]. In this part of the study, the general healing process as well as the macroscopic appearance and formation of scar tissue was assessed. Detailed photographic documentation was created after each step.

### 2.5. Histological Analysis

**Figure 2:**
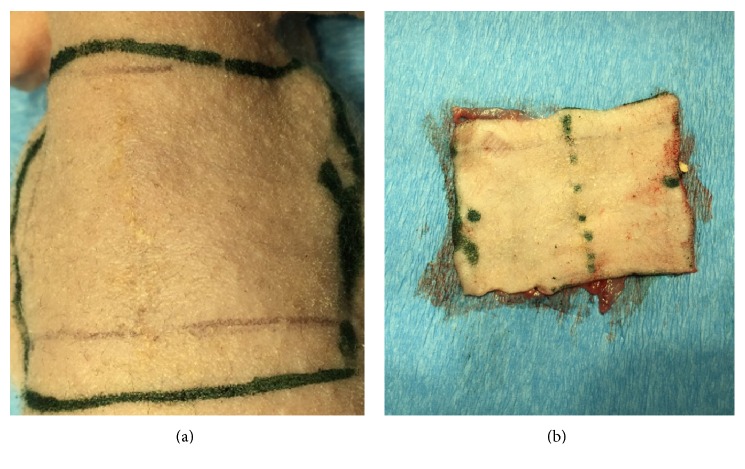
Preparation of specimens for histological, mechanical, and cell migration assays. (a) Marked dissection field before specimen preparation. (b) Derived rectangular 3 cm × 4 cm specimen of skin flaps containing subcutaneous tissues.

Just after the animals from the Study Group and all controls were sacrificed, the rectangular 3 cm × 4 cm skin flaps (Figures 2(a) and 2(b)) containing subcutaneous tissues were collected as specimens for standard histological analysis and assessment of the mechanical properties, using methods described in next section. From the prepared specimens, the 0.5 cm × 4 cm rectangular samples containing the line of healed wound were fixed in 10% buffered formalin for 24 h and processed for routine paraffin embedding. 5 *μ*m–thick sections were obtained from paraffin-embedded samples and stained with HE for further evaluation.

### 2.6. Mechanical Properties

The remaining 2.5 cm × 4 cm samples were examined with a Zwick & Roell Z0.5 machine to assess their mechanical properties. Tensile strength (tensometry) was examined using a specimen to test the skin-skin tissue union. Samples were cut into three similar segments and evaluated separately. The samples were fixed with tensometer clamps to place the healing line horizontally in the middle; stretching was performed with speed 250 mm/min to the breaking point. A 2.5 cm × 4 cm sample of healthy skin was taken from the posterior-dorsal field of one representative animal from each group. This specimen was compared with the experimental skin to determine the mechanical properties of each. Both specimens were examined by using the tensometer. The statistical differences between groups were calculated by ANOVA, followed by near infrared (NIR) post hoc multiple-comparison tests (IBM SPSS Statistics, Predictive Solutions, Poland). Statistically significant differences were defined as *p* < 0.05.

### 2.7. Cell Migration Assay

To assess potential cell migration of previously marked ADSCs, we used fluorochrome PKH26 markers. Skin, subcutaneous tissue, muscles (from the sheets directly adjacent to the ADSC injections and associated anatomical regions), thymus, brain, kidneys, lungs, liver, and spleen samples were collected just after all animals from Study Group were sacrificed. Tissues were frozen in an optimal cutting temperature compound (Tissue-TeK; Sakura, Torrance, California), and 5-*μ*m-thick sections were obtained. After rinsing with phosphate buffered saline (PBS), (pH = 7.4), the sections were fixed for 15 min with 4% paraformaldehyde (Sigma-Aldrich, St. Louis, MO, USA) in PBS. The cells were counterstained with DAPI (Sigma-Aldrich, St. Louis, MO, USA). The slides were then mounted in Aqua-Poly/Mount (Polysciences, Warrington, PA, USA) and examined using a C1 laser-scanning confocal microscope (Nikon, Tokyo, Japan) under 20% objective. The laser used for DAPI and PKH26 were diode 405 nm excitation with filter 450/35 and He-Ne 543 nm excitation with filter 650LP, respectively. The images were collected at the focal plane of brightest PKH26 fluorescence, using Nikon EZ-C1 3.80 software (Nikon Instruments, Melville, NY, USA). All images were generated using the same laser power, pixel dwell speed, and gain.

## 3. Results

**Figure 3:**
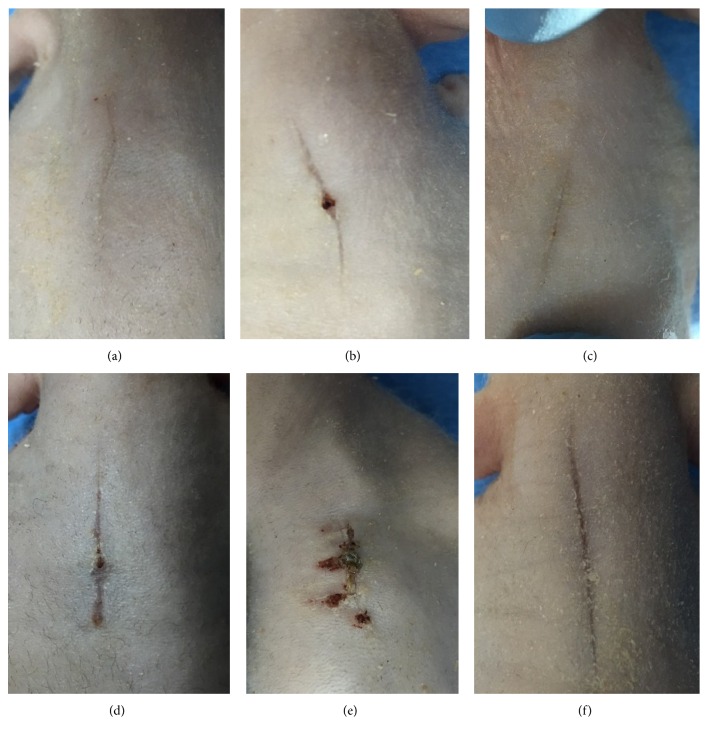
Cosmetic results observed in the assessment (performed after 1.5 months). (a) In the Study Group, linear, uncomplicated healing with symmetric flap union. (b) In C1 group, good healing with slight wound dehiscence. (c) In C2 group, linear healing without ruptures. (d) In C3 group, linear, uncomplicated healing usually with a small rupture in the middle of the wound. (e) In C4 group, healing with dry incrustation and slight tension. (f) In C5 group, easily visible scar formation and linear healing with symmetric, easily noticeable dehiscence.

**Figure 4:**
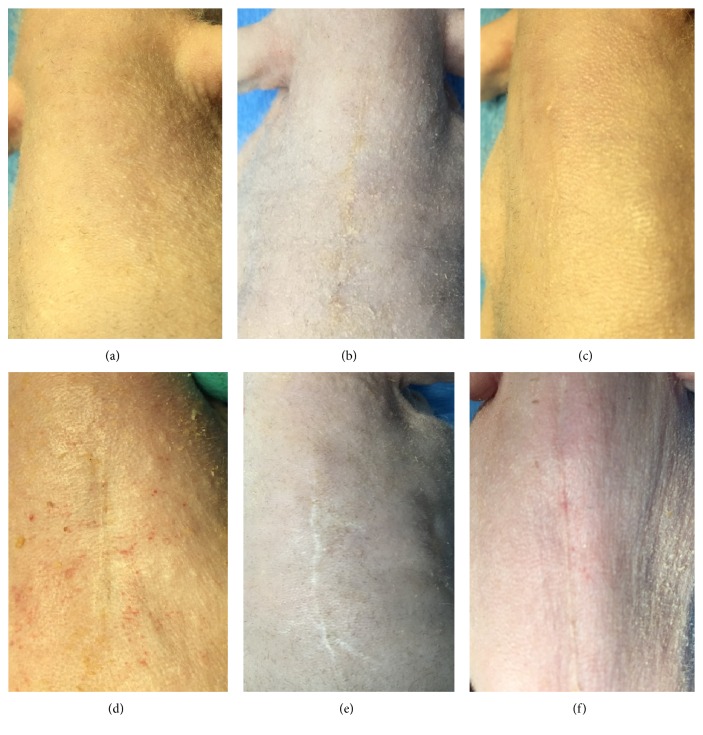
Final cosmetic results. (a) In Study Group, a mild, not palpable, linear, symmetric scar of esthetically favorable formation. (b) In C1 group, properly formed linear scar formation. In this group, minimal desquamation of dry epidermis was also observed. (c) In C2 group, a very thin, linear scar. (d) In C3 group, slight delving and very thin linear scar. (e) In C4 group, easily palpable and visibly asymmetric, nonlinear scarring corresponding to the course of sutures. (f) In C5 group, well palpable, protruding, and wide scarring.

All animals survived the planned 3-month study period. No pathological topical skin changes or bacterial contamination was observed. The wound healing process in all groups (Study Group and all controls alike) was uncomplicated, and no self-irritations or injuries were noted. In the macroscopic evaluation of cosmetic effects in the first assessment (performed after 1.5 months), all animals in Study Group exhibited linear, uncomplicated healing with symmetric flaps union (Figure 3(a)). Control group C1 exhibited good healing with slight wound dehiscence (Figure 3(b)). Control group C2 exhibited linear healing without ruptures (Figure 3(c)). Control group C3 exhibited linear, uncomplicated healing, usually with a small rupture in the middle of the wound course (Figure 3(d)). Control group C4 exhibited healing with dry incrustation and slight tension (Figure 3(e)). Control group C5 showed strongly visible scar formation and linear healing with symmetric, easily noticeable dehiscence (Figure 3(f)). After three months, all animals from every group exhibited a fully finished healing process. Final cosmetic effect observations were as follows. In Study Group, healing was characterized by mild but not palpable, linear, symmetric scar formation with good cosmetic appearance (Figure 4(a)). In group C1, properly formatted, linear scar formation was observed. In this group, minimal desquamation of dry epidermis was noted (Figure 4(b)). In C2 group, a very thin and linear scar was observed (Figure 4(c)). In C3 group, we observed slight delving and a very thin linear scar (Figure 4(d)). In C4 group, the scars were very palpable and characterized by a visible, asymmetric, nonlinear course corresponding to the course of sutures (Figure 4(e)). The scars formed in animals from the C5 group were well palpable, protruded, and wide (Figure 4(f)).

**Figure 5:**
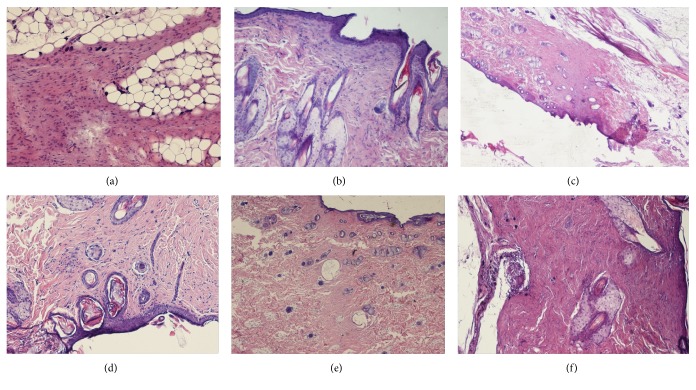
Histological assessment results. (a) Study Group scars were composed of dense collagen and spindle-shaped fibroblasts (proliferation of fibroblasts at the bottom of the scar) with preserved, singular vascular channels. Giant-cell, foreign-body type (residual features of the resorptive process) adipose tissue under the scar mummified/calcified cells mainly along the scar and blood vessels; magnification 2x. (b) In control group C1 scar tissue was composed of dense collagen and moderate numbers of spindle-shaped fibroblasts, with some mummified/calcified cells, mainly inside the scar (magnification 10x). (c) In the histological assessment of second control group C2, the scar tissue was composed of dense collagen with characteristic calcification of cells located especially in the central zone of the scar (magnification 2x). (d) In C3 group, the scar was composed of dense collagen and spindle-shaped fibroblasts with preserved, singular vascular channels and giant-cell, foreign-body type cells (residual features of the resorptive process), magnification 2x. (e) In C4 group, the scar was composed of dense collagen and spindle-shaped fibroblasts with preserved, singular vascular channels and hyperkeratosis of epidermis above the scar, magnification 2x. (f) In C5 group, the natural, untreated scar formation was composed of dense collagen and spindle-shaped fibroblasts with preserved singular vascular channels and foreign-body granuloma at the bottom of the scar, magnification 2x.

In the histological analysis of specimens derived from the animals in the Study Group, the scar was composed of dense collagen and spindle-shaped fibroblasts (proliferation of fibroblasts at the bottom of the scar) with preserved, singular vascular channels. In addition, giant-cell, foreign-body type (residual features of the resorptive process) adipose tissue under the scar produced mummified/calcified cells mainly along the scar and blood vessels (Figure 5(a)). In control group C1 scar tissue was composed of dense collagen and moderate numbers of spindle-shaped fibroblasts, with some mummified/calcified cells, mainly inside the scar (Figure 5(b)). In the histological assessment of second control group C2, the scar tissue was composed of dense collagen with characteristic calcification of cells located especially in the central zone of the scar (Figure 5(c)). The HE assessment of C3 group showed scarring composed of dense collagen and spindle-shaped fibroblasts with preserved, singular vascular channels of the giant-cell, foreign-body type (residual features of the resorptive process) (Figure 5(d)). In C4 group, scarring was composed of dense collagen and spindle-shaped fibroblasts with preserved singular, vascular channels and hyperkeratosis of epidermis above the scar (Figure 5(e)). In C5 group, the natural (unprotected wound) scar formation was composed of dense collagen and spindle-shaped fibroblasts with preserved, singular vascular channels and foreign-body granuloma at the bottom of the scar (Figure 5(f)).

**Figure 6:**
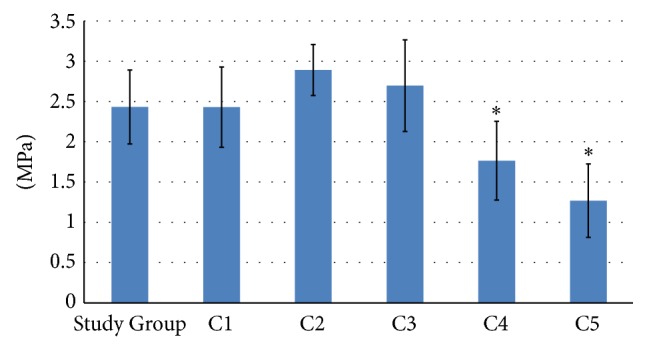
Presentation of statistical differences of mechanical property results among all groups. ^*∗*^Statistical significance was proven only in groups C4 and C5.

The mechanical properties analysis shows that the best tissue adaptability and strength were in group C2, in which the average breaking tension, measured in megapascals (MPa), was 2.890 MPa. A lower average was measured in C3 group—2.694 Mpa—than in Study Group—2.432 MPa. In the other groups, the following values were observed: in group C1: 2.430 MPa; in C4: 1.765 MPa; and in C5, the lowest 1.269 MPa. In the controlled, healthy (not manipulated) skin samples, the measured average was 13.036 MPa. The statistical significance was proven only in C4 and C5 groups (Figure 6).

**Figure 7:**
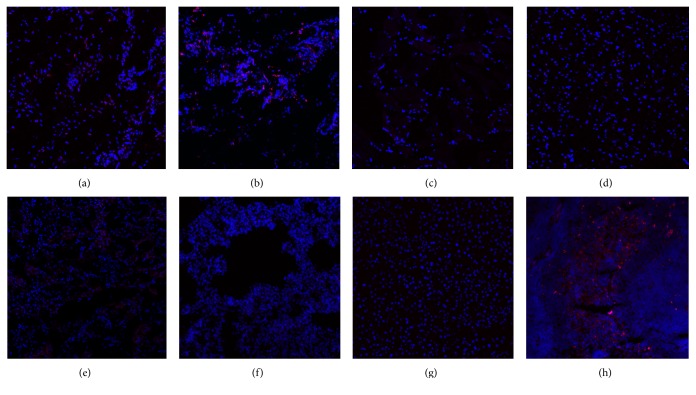
Cell migration assay using PKH26 fluorochrome. In all animals from Study Group, after 3 months, marked ADSCs were detected in the skin (a) and in subcutaneous tissue (b). Only a few cells showed positive staining in muscles surrounding the wound (c). A few marked cells and weak staining were found also in the brain (d). No sign of cell migration was detected in kidney (e), lung (f), and liver tissues (g). The highest fluorochrome PKH26 positive staining was observed in the spleen (h).

In the detailed cell migration assessment performed on all Study Group animals, using PKH26 marker and after three months, marked ADSCs were detected in the skin and in the subcutaneous tissue (Figures 7(a) and 7(b)). Only a few cells showed positive staining in muscles surrounding the wound model (Figure 7(c)). A smaller number of cells and weak staining also were found in the brain (Figure 7(d)). In contrast, no PKH26 staining was evident in kidney, lung, and liver tissues (Figures 7(e), 7(f), and 7(g)). The highest fluorochrome PKH26 positive staining was observed in the spleen (Figure 7(h)).

## 4. Discussion

In this paper, we present the novel use of adipose-derived stem cells to support topical adhesives to close artificial wounds in a rat model. Our study adds to current knowledge by assessing four main aspects of ADSC use in experimental wound closing: aesthetic outcome evaluation, histological analysis, mechanical properties evaluation, and cell migration assessment. The first evaluation point, general aesthetic outcome, is why we performed macroscopic cosmetic assessments twice (at 1.5 months and 3 months). A month and a half, halfway through the experiment, allowed us also to obtain important knowledge about the flow of the wound healing process and correspond it to standard clinical practice to detect early complications or address when a patient reports any inconvenience or dissatisfaction [24]. At this point in the study, no differences between first assessment and final results were reported in all groups that could be related to the rare clinical occurrence of deterioration when no early complications were detected [25].

The best cosmetic results were achieved in the Study Group and C2 group. In both groups, adhesive was used with supporting elements: in the Study Group with ADSCs and in C2 group with Steri-Strips. The better cosmetic results in animals from the Study Group could be from the potentially positive influence of ADSCs to initiate healing and the biologically friendly interaction with the skin milieu. This was reported in several in vivo projects in which it was proven that the use of this specific kind of cell improves skin grafts and directly promotes wound healing [22, 26]. The excellent aesthetic result achieved in group C2 is particularly encouraging. This could be likened to some clinical studies performed on humans in which Steri-Strips support wound healing by keeping optimal tension between wound surfaces; when that method is used along with adhesive use, it would be double wound protection [27]. This might also explain why in the groups that were closed only with adhesive (C1) or with only Steri-Strips (C3) the results were weaker. Weaker aesthetic results were also observed in C4 group, in which only standard sutures were used. This method has a statistically higher risk of unsatisfactory results than other closing methods [28]. The unacceptable results in C5 group, in which no closing method was used, are naturally associated with poor adaptation and wound healing [29].

Interesting data was obtained in the histological analysis besides the expected and well-known mechanisms described in literature, in which scar formation in control groups C2, C3, C4, and C5 corresponded to human clinical observations [30]. We also observed an unexpected spindle-shaped fibroblast proliferation at the bottom of the scar with preserved singular vascular channels and giant-cell, foreign-body type (residual features of the resorptive process) adipose tissue under the scar mummified/calcified cells mainly along the scar and blood vessels in the specimens from animals in the Study Group. This observation could correlate with the fact that the implemented cells were partly atrophied and died and with the widely discussed general problems of stem cell implementation related to the possibility of uncontrolled differentiation and proliferation [31]. In our study, no carcinogenic or other pathological changes were observed, but our findings do refer to some observations collected by Lambrou and Remboutsika that warn that such biological effects could occur in translational studies and should be monitored in studies with larger groups of animals. Multicenter analysis should be performed to confirm the full safety of stem cell application [32].

In the mechanical properties evaluation, high strength characteristics were presented in control groups C2 and C3; Steri-Strips were used in both groups and produced what could be considered better tissue surface adaptation and better strength control than with other wound-closing methods [33]. The best final median result was measured in C2 group, in which Steri-Strips (as in group C3) and adhesive were used to effect double prevention.

Next-best strength results, only minimally differing from C2 and C3 groups, were observed in the Study Group and C1 group. This situation confirms that in groups C4 and C5 the measured mechanical strength properties were much lower. No other studies have achieved the mechanical strength of natural (not previously injured) skin. The significant decrease of main mechanical properties in C4 and also in C5 group correspond to the currently published review and translational study data in which authors conclude that such decreased scar failure resistance would mainly suggest less total collagen in the tissue cross section due to either reduced size or number of collagen fibers. Such situation correspond also to the clinically noticed data in human in which the authors indicate that both the wound sutured and left without any supplies could not ever reach more than 3–50% of standard cutaneous tensile strength of normal uncut skin, mainly due to the significantly lower strength control, worse adaptation of tissues, and probably more negative influence of inflammatory response than when other wound-closing methods are used [34–36].

The last study point was cell migration aspects. In our study, we confirmed that ADSCs remain active and function in the skin like subcutaneous tissue. The most interesting finding is the detection of some marked cells in muscles surrounding the wound, which confirm the high regenerative potential of ADSCs. The puzzling detail of the study is the detection of minimal, nonrepresentative cell numbers in brain tissue specimens, which has also been reported in other basic and preclinical studies such as the natural cell migration to the spleen when using other types of cells [37].

## 5. Conclusions

In our study, we proved that ADSCs could be used effectively to support topical skin adhesives to close wounds for better cosmetic and aesthetic outcomes. In our opinion, additional studies should be performed to define the specific role, safety, expected benefits, and limitations of ADSC use to support wound closure with standard topical adhesives.
